# Comparative Transcriptomic and Physiological Analyses of *Medicago sativa* L. Indicates that Multiple Regulatory Networks Are Activated during Continuous ABA Treatment

**DOI:** 10.3390/ijms20010047

**Published:** 2018-12-22

**Authors:** Dong Luo, Yuguo Wu, Jie Liu, Qiang Zhou, Wenxian Liu, Yanrong Wang, Qingchuan Yang, Zengyu Wang, Zhipeng Liu

**Affiliations:** 1 The State Key Laboratory of Grassland Agro-ecosystems, Key Laboratory of Grassland Livestock Industry Innovation, Ministry of Agriculture, College of Pastoral Agriculture Science and Technology, Lanzhou University, Lanzhou, 730020, China; luod13@lzu.edu.cn (D.L.); wuyg16@lzu.edu.cn (Y.W.); liuj18@lzu.edu.cn (J.L.); zhouq2013@lzu.edu.cn (Q.Z.); liuwx@lzu.edu.cn (W.L.); yrwang@lzu.edu.cn (Y.W.); 2 Institute of Animal Sciences, Chinese Academy of Agricultural Sciences, Beijing 100000, China; qchyang66@163.com; 3 Core Research & Transformation, Noble Research Institute, Ardmore, OK 73401, USA

**Keywords:** ABA treatment, alfalfa, antioxidative defense, pathogen immunity, differentially expressed isoforms, physiological shifts

## Abstract

Alfalfa is the most extensively cultivated forage legume worldwide. However, the molecular mechanisms underlying alfalfa responses to exogenous abscisic acid (ABA) are still unknown. In this study, the first global transcriptome profiles of alfalfa roots under ABA treatments for 1, 3 and 12 h (three biological replicates for each time point, including the control group) were constructed using a BGISEQ-500 sequencing platform. A total of 50,742 isoforms with a mean length of 2541 bp were generated, and 4944 differentially expressed isoforms (DEIs) were identified after ABA deposition. Metabolic analyses revealed that these DEIs were involved in plant hormone signal transduction, transcriptional regulation, antioxidative defense and pathogen immunity. Notably, several well characterized hormone signaling pathways, for example, the core ABA signaling pathway, was activated, while salicylic acid, jasmonate and ethylene signaling pathways were mainly suppressed by exogenous ABA. Moreover, the physiological work showed that catalase and peroxidase activity and glutathione and proline content were increased after ABA deposition, which is in accordance with the dynamic transcript profiles of the relevant genes in antioxidative defense system. These results indicate that ABA has the potential to improve abiotic stress tolerance, but that it may negatively regulate pathogen resistance in alfalfa.

## 1. Introduction

Cultivated alfalfa (*Medicago sativa* L.) is a perennial, cross-pollinated, autotetraploid (2n = 4x = 32) plant with a genome size of 800–900 Mb [1]. This species is referred to as the “queen of forages”, and is used as hay, silage and pasture for ruminant animals and in dairy production [2]. Moreover, alfalfa has considerable potential as a biofuel feedstock for ethanol production [3]. Biorefining could enhance the return of alfalfa production so that cultivation of this Leguminosae crop is more economically attractive as well as environmentally beneficial [4]. In China, alfalfa plantation areas are distributed in 14 provinces throughout the Northern region of the country. However, in these areas, abiotic and biotic stresses are two major environmental constraints that impact the alfalfa’s quality and yield, causing severe production losses. Recent studies indicated that phytohormone abscisic acid (ABA) regulates numerous key processes in plants and serves as a pivotal endogenous messenger in abiotic and biotic stresses [5,6]. Therefore, an improved understanding of the basic mechanisms of plant responses to ABA is important for breeders to improve the abiotic and biotic stress tolerances of alfalfa.

Abiotic stress (such as cold, drought, salinity and heat) results in strong increases of ABA level, and this accumulation promotes stress tolerance in various plant species [7,8]. Likewise, it was also shown that exogenous ABA application enhanced plant abiotic stress tolerance through a series of physiological and biochemical changes. For example, research on bermudagrass (*Cynodon dactylon*) has shown that pretreatment with ABA mimic 1 increased bermudagrass cold stress tolerance through the modulation of reactive oxygen species (ROS) level, antioxidant enzyme activities and osmolyte contents [9]. Gurmani et al. (2011) showed that rice (*Oryza sativa*) seeds presoaked with ABA developed enhanced salinity tolerance through the suppression of Na^+^ and Cl^+^ levels, lowering Na^+^/K^+^ ratios as well as increasing soluble sugar content [10]. Previous studies have also shown that under drought conditions exogenous ABA application enhanced the growth and quality of creeping bentgrass (*Agrostis Stolonifera*), Kentucky bluegrass (*Poa pratensis*) and tall fescue (*Festuca arundinacea*). This is associated with a reduction in water loss and increased osmotic adjustment [11].

ABA involves a physiological response to abiotic stress as well as biotic stress. Mounting evidence suggests that ABA appears to act as either a synergistic or an antagonistic regulator during defense responses to biotic stress [12]. For example, it was reported that ABA is associated predominantly with pathogen susceptibility in tomato (*Solanum lycopersicum*) [13], soybean (*Glycine max*) [14] and rice [15] crops. ABA treatment increased the susceptibility of these plants mainly through suppressing the phytoalexin synthesis and inhibiting the activity of phenylalanine ammonium lyase [16]. However, in *Arabidopsis*, ABA has been shown to be a positive regulator of disease resistance and ABA-activated stomatal closure is a key barrier against pathogens infection [17]. Furthermore, the reasons for the different physiological effects of ABA in biotic stress resistance were also revealed. This may depend on the type of pathogen, pathogen lifestyle and mode of pathogen attack, as well as the concentration of ABA [18].

The physiological responses of ABA during abiotic and biotic stresses are all initiated upon the activation of molecular networks within signaling pathways. Molecular analyses have demonstrated the existence of ABA-dependent signal transduction cascades that occur between an initial stress signal and the expression of specific genes [19]. It is well known that the abiotic stress signaling pathway involves a core ABA-signaling module: pyrabactin resistance 1-like (PYL)–protein phosphatase 2C (PP2C)–the sucrose nonfermenting1-related protein kinase 2 (SnRK2). Under abiotic stress, the PYL–PP2C–SnRK2 core ABA-signaling module is activated by abiotic stress signals and then specifically binds to ABA-responsive elements (ABREs) called ABRE binding factors (ABFs), which ultimately promotes the expression of downstream ABA/stress-responsive genes [20]. In contrast, ABA-inducible pathogen defenses involve a salicylic acid (SA)–jasmonate (JA)–ethylene (ET) signaling pathway. In this signaling system, ABA acts synergistically or antagonistically with SA–JA–ET signaling, creating a plant-immune signaling network of interacting pathways with cross-talk at different levels [21]. Therefore, a different molecular response of ABA exists between abiotic and biotic stress.

Considering its complexity, it is essential to interpret the functional elements and molecular constituents involved in ABA-responsive mechanisms on a whole-genome level in plants. Using microarray technology, a large number of ABA-responsive genes have been identified in many plant species including sorghum (*Sorghum bicolor*) [22], cotton (*Gossypium hirsutum*) [23] and rice [24]. Given the relatively inaccurate characteristics of microarray-based approaches, the recently developed high-throughput RNA sequencing (RNA-Seq) has become the ideal option to be used in gene discovery and regulatory network studies [25]. Based on RNA-Seq platforms, genome-scale transcriptome analyses were used to identify ABA-responsive genes in tomato [18], strawberry (*Fragaria ananassa*) [26] and peanut (*Arachis hypogaea*) [27] crops. These ABA-responsive genes identified by RNA-Seq are involved in many physiological and metabolic processes, such as protection against cell wall toxicity and oxidative stress, pathogen defense, hormone signal transduction and compatible solute metabolism. However, so far, genome-wide transcriptomic analysis of the ABA-responsive genes in alfalfa has not been reported, especially within the root tips, which are the primary sites for the perception of ion toxicity, high osmotic pressure and soil-borne pathogen attack [28,29]. Thus, in this study, we carried out the first global transcriptome analysis of alfalfa root tips, which were treated with 10 µM ABA for 0, 1, 3 and 12 h using the BGISEQ-500 RNA-Seq platform. Reference sequences were derived from our previous study of a PacBio full-length transcriptome database of alfalfa. The physiological effects of ABA on cell damage and ROS accumulation, as well as underlying antioxidant and osmoprotectant responses, were also determined. The results of this study will expand our knowledge of the response of alfalfa roots to ABA and will also provide a novel genetic resource for breeders to improve the abiotic and biotic stress tolerances of alfalfa.

## 2. Results

### 2.1. Physiological Responses to ABA

**Figure 1:**
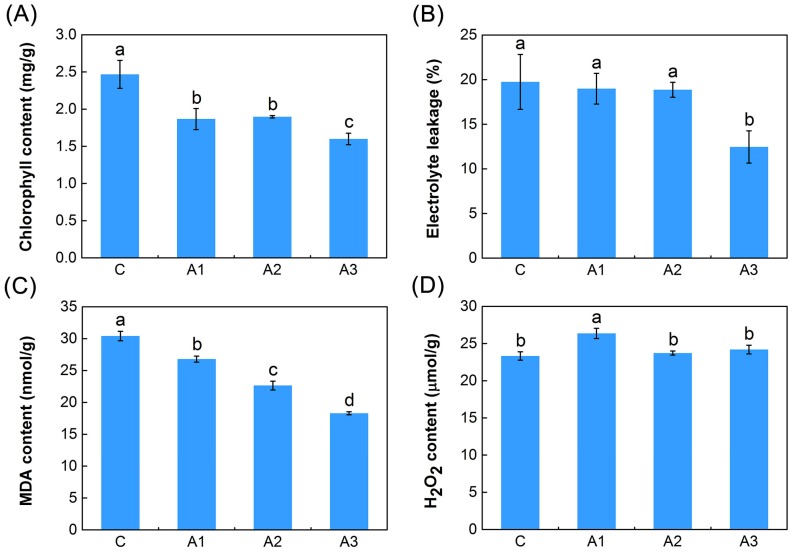
Analyses of dynamic physiological effects under continuous ABA treatment. (**A**) Chlorophyll content. (**B**) Electrolyte leakage. (**C**) Malonaldehyde (MDA) content. (**D**) Hydrogen peroxide (H_2_O_2_) content. C represent control; A1, A2 and A3 represent ABA treatment for 1, 3 and 12 h, respectively. The results are the means and standard deviation (SDs) of the three replicates. Different letters above the bars indicate significant difference treatments (Duncan’s multiple range test; *p* < 0.05).

The electrolyte leakage level, chlorophyll contents and malonaldehyde (MDA) content were determined to evaluate the cell membrane stability, photosynthesis ability and the extent of cell damage of alfalfa under ABA treatment, respectively. In this study, a decrease in chlorophyll content was observed in the three groups (1 h (A1), 3 h (A2) and 12 h (A3)) under ABA treatment, compared with the control (C) group, and showed significant differences (*p* < 0.05) (Figure 1A). As shown in Figure 1B, the levels of electrolyte leakage remained stable in the first two groups (A1 and A2) under ABA treatment, but later they decreased significantly (*p* < 0.05) in the A3 group compared with the C group. Moreover, as shown in Figure 1C, ABA treatment caused significant decreases (*p* < 0.05) in MDA content in the three groups (A1, A2 and A3) compared with the C group, and the changes became more evident with increasing time.

Since ABA can cause an increased generation of ROS such as hydrogen peroxide (H_2_O_2_), the effects of 10 µM ABA on levels of H_2_O_2_ in the roots of the alfalfa seedlings were investigated. As shown in Figure 1D, there were no evident differences in the H_2_O_2_ levels between the two ABA treatment groups (A2 and A3) and the C group, but the H_2_O_2_ levels increased significantly (*p* < 0.05) in the A1 group.

**Figure 2:**
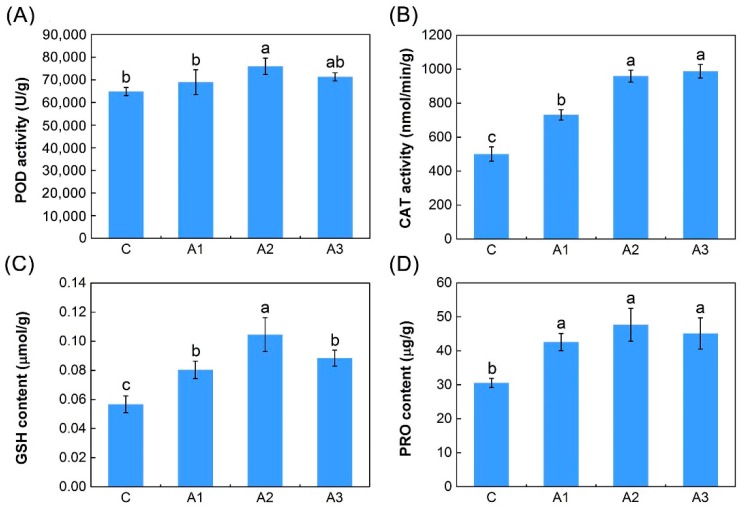
Analyses of dynamic physiological responses under continuous ABA treatment. (**A**) Peroxidase (POD) activity. (**B**) Catalase (CAT) activity. (**C**) Glutathione (GSH) content. (**D**) Proline (PRO) content. C, A1, A2 and A3 represent ABA treatment for 0, 1, 3 and 12 h, respectively. The results are the means and standard deviation (SDs) of the three replicates. Different letters above the bars indicate significant difference treatments (Duncan’s multiple range test; *p* < 0.05).

To evaluate whether the cellular antioxidant defense system was activated, the activities of key antioxidant enzymes, such as peroxidase (POD) and catalase (CAT), and the contents of antioxidants, such as reduced glutathione (GSH), were tested. The POD activity increased when alfalfa was exposed to ABA treatment conditions (Figure 2A). Compared with plants in the C group, a significant difference (*p* < 0.05) was observed only in A2. In parallel with the POD activity, a significant increase (*p* < 0.05) in CAT activity was observed in three groups (A1, A2 and A3) under ABA treatment compared with the C group, and a steady trend appeared after A2 (Figure 2B). Similarly, the measured GSH contents were higher in all three ABA-treated samples compared with the C group, and showed significant differences (*p* < 0.05) (Figure 2C). Additionally, because proline (PRO) performs a protective function by scavenging ROS, leading to enhanced antioxidant defense systems, the content of PRO was also examined. The PRO content of alfalfa roots increased markedly during the 12-h ABA treatment group, which showed similar patterns to that of GSH activity (Figure 2D).

### 2.2. Transcriptome Sequencing, Assembly and Annotation

To achieve greater global and comprehensive coverage of the gene expression profiles of alfalfa roots under ABA treatment, 12 cDNA libraries receiving one control (C, without ABA treatment) and three ABA treatments at different time points (A1, A2 and A3) were designed for high-throughput RNA-Seq; each of the four treatment groups involved three biological replicates. A total of 272,487,657 raw reads were ultimately obtained (Table S1). After removing the redundancy, a total of 265,901,178 high-quality clean reads remained, constituting over 13.3 GBase, with each library constituting more than 1.0 GBase (Table S1). Using the Bowtie2 software, an average of 72.90% of clean reads were uniquely mapped to the alfalfa full-length transcripts (Table S1). As a result, a total of 50,742 isoforms were assembled, and a total of 47,239, 47,632, 47,679 and 47,260 isoforms were identified for the C, A1, A2 and A3 groups, respectively (Table S2). The length of these 50,742 isoforms ranged from 303 to 8445 bp, with an N50 and N70 length of 2246 and 3017 bp, respectively (Figure S1A). The length distribution of the isoforms in each of the 12 libraries is shown in Figure S1B. All the sequence read data were deposited in the National Center for Biotechnology Information (NCBI) Sequence Read Archive database (SRR7160313, 16–21, 39–41, 50, 53, one number for each library).

For the functional annotation of all the transcripts, BLASTx (*E*-value ≤ 10^−5^) searches were carried out to perform functional annotations with transcripts against public databases, including the NCBI non-redundant protein sequences (Nr), Gene Ontology (GO) and Kyoto Encyclopedia of Genes and Genomes (KEGG) databases. Of these 50,742 all-isoforms, 50,041 (98.62%), 15,031 (29.62%) and 40,741 (80.29%) isoforms were successfully annotated in the Nr, GO and KEGG databases, respectively; a total of 50,050 (98.64%) and 13,830 (27.26%) isoforms were annotated in at least one database and in all databases, respectively (Table S2).

### 2.3. Verification of Gene Expression

**Figure 3:**
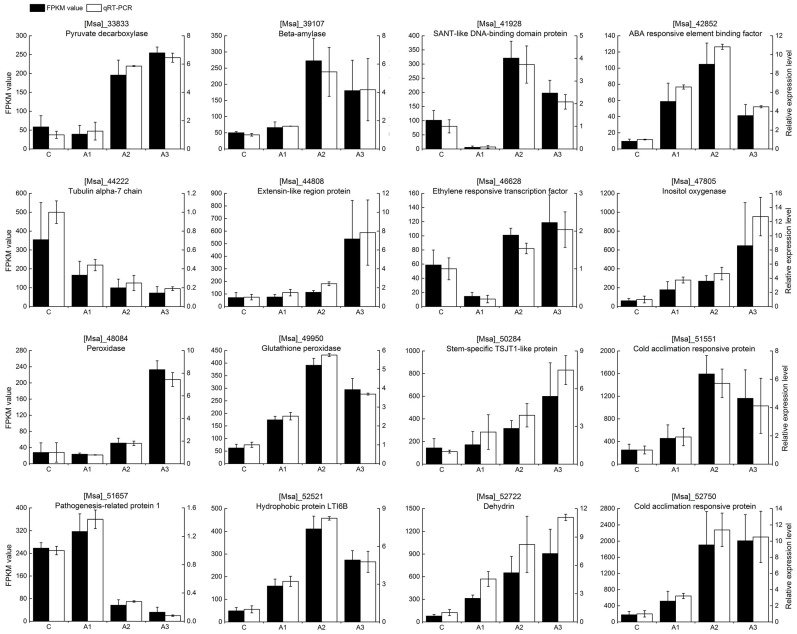
The expression pattern of 16 randomly selected genes identified by RNA-Seq, as verified by qRT-PCR. C, A1, A2 and A3 represent ABA treatment for 0, 1, 3 and 12 h, respectively. Black bars represent the transcript abundance change based on the fragments per kilobase per million fragments mapped (FPKM) values of the RNA-Seq analysis (left *y*-axis). White bars indicate the relative expression levels measured by qRT-PCR (right *y*-axis). Error bars indicate the standard errors of the means (*n* = 3).

To confirm the reliability of our transcriptome data, 16 isoforms were randomly selected for quantitative real-time polymerase chain reaction (qRT-PCR) validation. In our analysis, the expression profiles of these 16 isoforms determined by qRT-PCR were consistent with the RNA-seq data (Figure 3), thus indicating that our RNA-seq data was accurate and reliable and could be used to identify transcripts that are differentially regulated in response to ABA.

### 2.4. Differentially Expressed Isoforms (DEIs) Analysis

**Figure 4:**
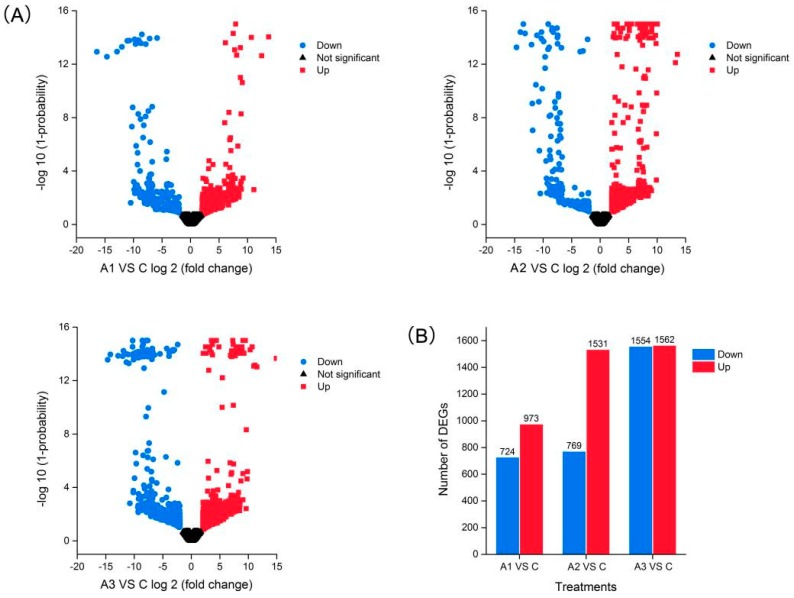
Identification of the DEIs in response to ABA deposition. (A) Volcano plots display log_2_ (fold change) and log_10_ (1-probability) values. (B) The number of upregulated and downregulated DEIs at each treatment time point compared with the control. C, A1, A2 and A3 represent ABA treatment for 0, 1, 3 and 12 h, respectively.

Upon comparison with the control group, the isoforms that met the default criteria with an absolute value of fold change ≥4 and a divergence probability ≥0.8 as found by NOISeq software were assigned as differentially expressed isoforms (DEIs). Based on these strict criteria, a total of 1697 (973 upregulated and 724 downregulated), 2300 (1531 upregulated and 769 downregulated) and 3116 (1562 upregulated and 1554 downregulated) DEIs were found to respond to ABA in the A1, A2 and A3 groups, respectively (Figure 4A,B), indicating that ABA treatment caused significant changes in gene expression in alfalfa roots. The number of upregulated DEIs increased dramatically from A1 to A2 and then maintained steady at A3, while the number of downregulated DEIs remained nearly constant between A1 and A2 and showed a pronounced decrease at A3. Furthermore, a total of 4944 DEIs were detected after 12 h ABA treatment, and 526 were common to all three time points, suggesting that these genes were continuously significantly modulated during the 12 h ABA treatment (Figure S2). There were 697, 846 and 1758 DEIs specifically modulated in A1, A2 and A3, which represented early, medium and late responsive DEIs, respectively (Figure S2).

**Figure 5:**
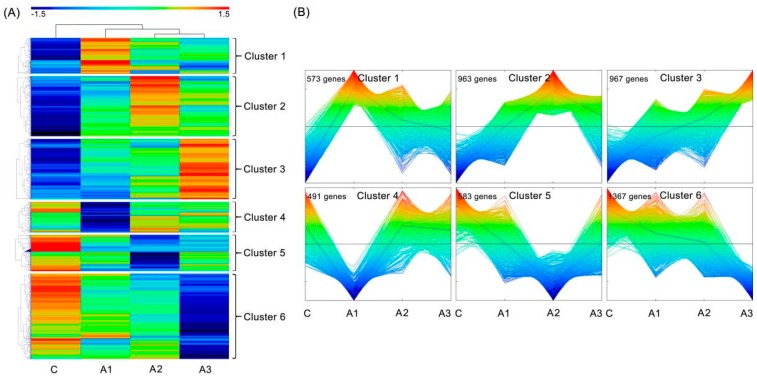
An overview of continuous dynamic changes in DEI expression levels. (**A**) Heatmap showing all DEIs using the MEV 4.9 software with the hierarchical clustering method; six clusters are shown. (**B**) The dynamic expression of the DEIs in each of the six clusters was analyzed via MEV4.9 software with the K-means clustering method. C, A1, A2 and A3 represent ABA treatment for 0, 1, 3 and 12 h, respectively. The gene expression is based on the z-scores of log_2_ (FPKM) value. The red and blue colors indicate high and low expression levels, respectively.

Using MultiExperiment Viewer 4.9 (MEV 4.9) software, all 4944 DEIs were classified into six clusters with the hierarchical clustering algorithm (Figure 5A). The dynamic expression of the DEIs in each cluster was then analyzed via MEV4.9 software with the K-means clustering algorithm (Figure 5B). These six clusters were divided into three upregulated patterns (Clusters 1, 2 and 3) and three downregulated patterns (Clusters 4, 5 and 6) with highly similar temporal expression patterns. The expression level of Clusters 1, 2 and 3 peaked at A1, A2 and A3, respectively, while that of Clusters 4, 5 and 6 reached the minimum at A1, A2 and A3, respectively.

### 2.5. GO Enrichment Analysis of the DEIs

**Figure 6:**
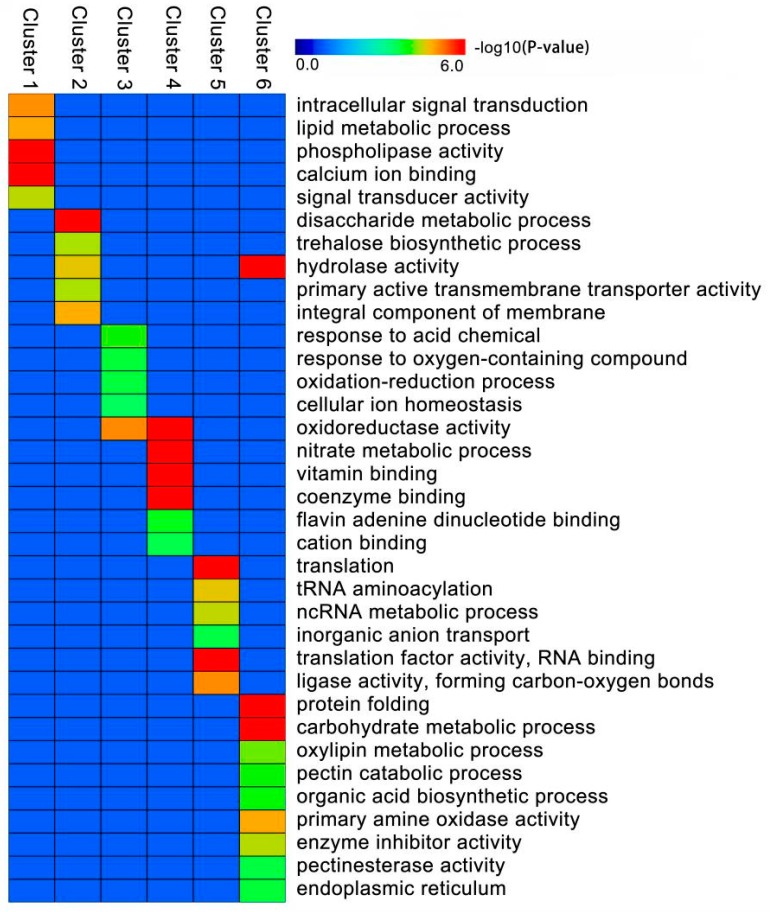
GO function enrichment analysis of different clusters. The names of the GO categories are listed along the *y*-axis. The degree of GO enrichment is represented by the log_10_ (*p*-value). The log_10_ (*p*-value) value ranged from 0 to 6; a log_10_ (*p*-value) closer to 6 indicates greater enrichment.

We applied GO category enrichment analysis for functional significance of all 4944 DEIs with six clusters activated under ABA treatment (Figure 5) using the agriGO 2.0 website. The groups of genes in each cluster showed confident enrichments for particular functional categories (Figure 6). In general, different functional categories were enriched between the upregulated DEIs (Clusters 1, 2 and 3) and the downregulated DEIs (Clusters 4, 5 and 6). Clusters 1–3 were mainly involved in signal transduction, transmembrane transporter, phospholipase activity and cellular ion homeostasis. In contrast, Clusters 4–6 were mainly associated with binding, translation, protein folding and enzyme inhibitor activity. A few common functional categories were also observed between the upregulated DEIs and the downregulated DEIs. Clusters 1–3 and Clusters 4–6 were commonly related to two functional categories, including oxidoreductase activity and hydrolase activity.

### 2.6. Identification of Transcription Factors (TFs) of the DEIs

**Figure 7:**
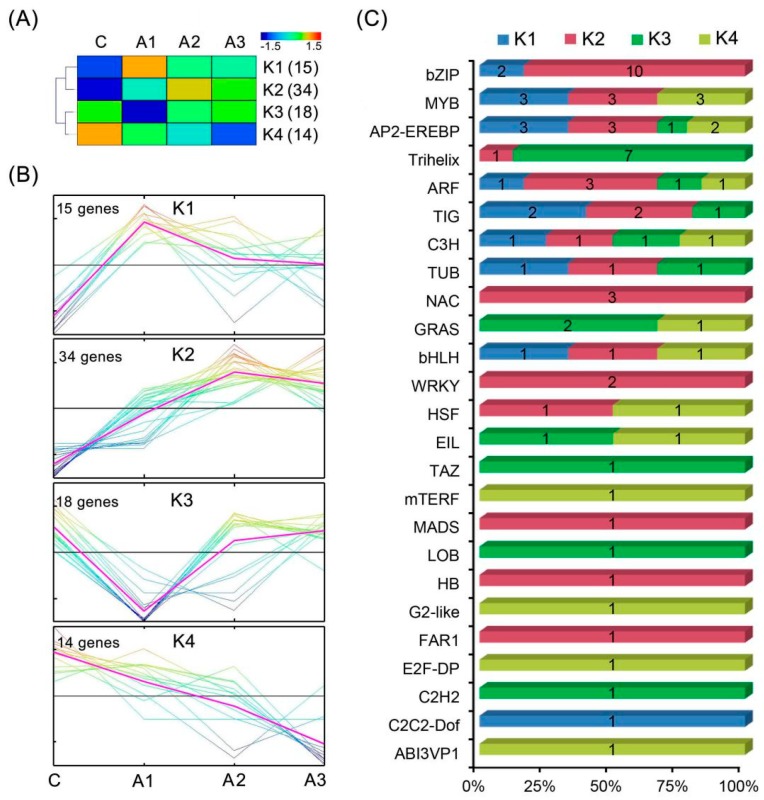
Dynamics of transcription factor accumulation profiles. (**A**) All significantly differentially expressed TFs from all analyzed ABA-treated time points were clustered into four lineages (K1–4) using the MEV 4.9 software with the Self-Organizing Tree Algorithm (SOTA) method; (**B**) The dynamic expression of the TFs in each of the four clusters was analyzed using the MEV4.9 software with the K-means clustering method. (C) The distribution of TFs in each TF family among K1–4. C, A1, A2 and A3 represent ABA treatment for 0, 1, 3 and 12 h, respectively.

In response to ABA deposition, the alfalfa transcriptome includes the altered expression of transcripts encoding transcription factors (TFs). Of the 4944 DEIs, 82 belong to 25 TF families (Table 1). Members of the bZIP family were the most abundant (*n* = 12), followed by the MYB (*n* = 9), AP2/EREBP (*n* = 9), Trihelix (*n* = 8) and ARF (*n* = 6) families. Based on the Self-Organizing Tree Algorithm (SOTA) in the MEV4.9 program, the expression patterns of differentially expressed TFs were clustered into four groups (designated K1–4, Figure 7). Our data showed that the members of seven TF families showed clear upregulated expression patterns (K1 and K2) under ABA treatment, such as bZIP, NAC, WRKY and MADS, while the members of nine TF families showed consistently downregulated expression patterns (K3 and K4), such as GRAS, ABI3VP1, EIL and LOB. In contrast, the members of the remaining nine TF families showed differently regulated expression patterns, such as MYB (six upregulated and three downregulated), AP2-EREBP (six upregulated and three downregulated), Trihelix (one upregulated and seven downregulated) and ARF (four upregulated and two downregulated).

### 2.7. KEGG Pathway Enrichment Analysis of the DEIs

**Figure 8:**
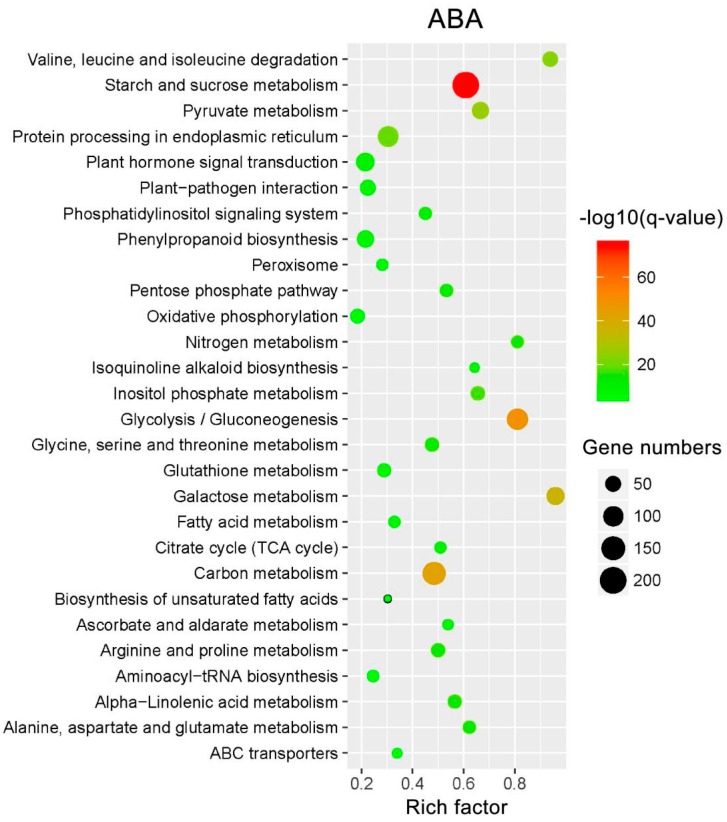
Scatterplot of enriched KEGG pathways for DEIs. The DEIs from all analyzed ABA-treated time points. The rich factor is the ratio of the DEI number to the total gene number in a certain pathway. The size and color of the dots represent the gene number and the range of the log_10_ (*q*-value), respectively.

To understand the complex biological behaviors of the transcriptome profiles, KEGG pathway enrichment analyses were performed on all DEIs using the KOBAS 3.0 website. A total of 4046 ABA-responsive DEIs were assigned to 119 different KEGG pathways, and 28 pathways changed significantly (*q*-value < 0.05) under ABA treatment (Figure 8). The significantly over-represented pathways were mainly involved in multiple signaling, biosynthesis and metabolism processes, including ‘‘plant hormone signal transduction‘‘, ‘‘phosphatidylinositol signaling system‘‘, ‘‘ABC transporters‘‘, ‘‘biosynthesis of unsaturated fatty acids‘‘, ‘‘isoquinoline alkaloid biosynthesis‘‘, ‘‘peroxisome‘‘, “starch and sucrose metabolism”, ‘‘arginine and proline metabolism‘‘ and ‘‘plant-pathogen interaction”.

## 3. Discussion

ABA, as a key phytohormone, plays a vital role in plant stress response [30]. ABA and stress treatments have been found to extensively change the expression level of plant genes [31]. As a globally important forage legume, alfalfa is defined as a moderately sensitive crop to stress. Dissecting the molecular mechanism underlying the ABA response in alfalfa will be helpful for us to develop new alfalfa cultivars that are tolerant to stresses. Consequently, the present study reports the comprehensive transcriptional response of alfalfa root tips under ABA treatment for 1, 3 and 12 h. A total of 50,742 isoforms were generated, and 4944 DEIs were identified after ABA deposition. Metabolic analyses revealed that these DEIs were involved in plant hormone signal transduction, transcriptional regulation, antioxidative defense and pathogen immunity. These processes might contribute to the recovery after ABA deposition on alfalfa, and the related DEIs merit further study.

### 3.1. ABA Regulation Pathway-Related DEIs

**Figure 9:**
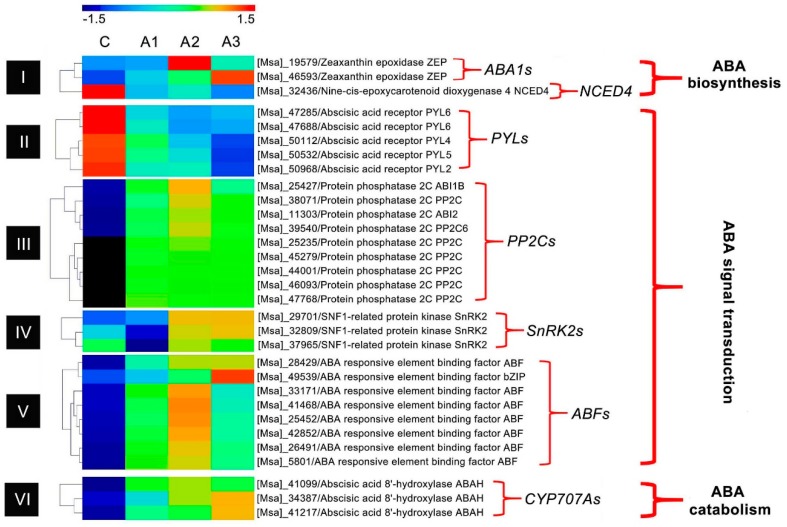
Transcriptional expression of ABA signal core components. C, A1, A2 and A3 represent ABA treatment for 0, 1, 3 and 12 h, respectively. Heat map showing all DEIs involved in the ABA regulatory pathway using the MEV4.9 software with the hierarchical clustering method. The gene expression is based on the z-scores of log_2_ (FPKM) value. The red and blue colors indicate high and low expression levels, respectively.

External applications of ABA can partly compensate for deficiencies in endogenous ABA, which is determined by the rate of ABA biosynthesis and catabolism in plants [32,33]. In the present transcriptome analysis, two genes encoding for zeaxanthin epoxidase (ZEP/ABA1), which catalyzes the initial step of ABA biosynthesis, showed increased expression at all ABA-treated time points (Figure 9 and Figure S3) [31]. Interestingly, the genes involved in the later steps of ABA biosynthesis, which are induced in other plants under abiotic stress, such as *xanthoxin dehydrogenase* (*XanDH/ABA2*), *abscisic-aldehyde oxidase* (*AAO*) and *molybdenum cofactor sulfurase* (*MCSU/LOS5/ABA3*) [34], were not found. One gene belonging to the *9-cis-epoxycarotenoid dioxygenase 4* (*NCED4*), which was the key gene in norisoprenoid metabolism leading to the biosynthesis of ABA [35], was identified, but its transcript abundance was downregulated (Figure 9 and Figure S3). Meanwhile, transcripts for three ABA 8′-hydroxylase (*ABAH/CYP707A*) genes which have been widely reported to function as an enzyme that catalyzes ABA catabolism [36], were identified, and showed increased expression at all ABA-treated time points (Figure 9 and Figure S3). These results suggest that exogenous ABA may affect both the ABA biosynthesis and catabolism in alfalfa roots, but the regulatory mechanism is diverse and complex. Therefore, further exploration is needed to understand the detailed regulatory mechanism.

Current evidence suggests the existence of ABA-dependent and ABA-independent signal transduction cascades that occur between an initial stimulus signal and the expression of target genes in plants [19]. ABA-dependent signal transduction is mainly regulated by four core regulatory components: PYLs, PP2Cs, SnRK2s and ABFs [37]. In the presence of ABA, PYL receptors bind to ABA and prevent the PP2C-mediated dephosphorylation of SnRK2, causing the activation of SnRK2 kinases. Active SnRK2s can phosphorylate bZIP group TFs, such as ABFs, which then promote the downstream ABA-responsive gene transcription, thus eliciting ABA responses [38]. In this study, all of the key genes involved in ABA signaling mentioned above were identified. The transcript abundances for five *PYLs* were all reduced during the 12 h treatment, whereas the remaining genes, including nine *PP2Cs*, three *SnRK2s* and eight *ABFs* were uniformly upregulated after treatment with ABA for 12 h (Figure 9 and Figure S3). These results agree with what is known of the ABA regulation pathway [31], indicating that exogenous ABA can activate the ABA signaling pathway and that it affected PYLs, PP2Cs, SnRK2s and ABFs.

### 3.2. Transcriptional Regulation-Related DEIs

TFs are crucial components in plant hormone-mediated regulation of plant growth, development and stress responses, which are positioned at the penultimate step in signal cascade and directly control downstream target gene expression [39]. Within the alfalfa transcriptome, at least 82 TFs in 25 TF families were identified as DEIs during the ABA treatment, such as the *bZIP*, *AP2/EREBP*, *MYB*, *Trihelix* and *ARF* TF families (Figure 7, Table 1 and Table S3).

The bZIP family is one of the largest TF families in plants, and its members have diverse roles, particularly in organic differentiation, stress response and hormone signal transduction [40]. Researchers have shown that a bZIP-type *SlAREB* from tomato can bind to ABRE, thereby activating ABA-responsive genes *AtRD29A*, *AtCOR47* and *SlCI7-like dehydrin*; additionally, the overproduction of *SlAREB* in *Arabidopsis* and tomato plants has been shown to be able to improve tolerance to water deficit and salinity stress [41]. In this study, the largest class of TFs induced by ABA treatment was the *bZIP* TF family, including eight *ABF/AREB* TFs and four G-box-binding *bZIP* TFs. All 12 members of the *bZIP* TF family were upregulated either at the early stage (A1) or at a later stage (A2 or A3) of alfalfa ABA treatment (Figure 7C), suggesting that bZIP TFs were probably positive regulators of ABA signaling in alfalfa.

The AP2/EREBP superfamily is a large group of plant-specific TFs that includes four major subfamilies: the AP2, DREB, ERF and RAV TF subfamilies [42]. Of these, AP2 TFs have diverse functions in crown root initiation and ABA-driven cellular responses and tolerance to abiotic stresses, while ERF TFs encode multifunctional factors that integrate various signal transductions, such as ABA and the JA/ET signaling pathways, and thus potentially play dual roles in abiotic and biotic stresses in plants [43]. In this study, nine DEIs were identified as *AP2/EREBP* TFs, including four *AP2* TFs and five *ERF* TFs. Among these TFs, the transcript abundances for three of the four *AP2* TFs and three of the five *ERF* TFs were upregulated, whereas the remaining genes were downregualted (Figure 7C), which suggests a high biological importance of AP2/EREBP TFs in response to ABA deposition in alfalfa.

The MYB, Trihelix and ARF TF families are also involved in the ABA-dependent regulatory systems of plants responding to environmental stresses [44,45,46]. These TFs showed both inducible and suppressed expression patterns after ABA deposition (Figure 7C), suggesting that sophisticated transcriptional regulation could participate in alfalfa response to ABA and adverse environments. These results are similar to those of a study of tomato, which reported that various TFs belonging to several classes, including *bZIP*, *bHLH*, *MYB*, *AP2/ERF*, *NAC* and *WRKY* TFs, engaged in ABA-mediated gene expression [18].

### 3.3. Antioxidant Defense System-Related DEIs

Previously, ABA has been implicated in enhancing tolerance to abiotic stress in various plant species [9,10,11], but currently little is known about the molecular mechanisms underlying this phenomenon. One possible model is that ABA-mediated metabolic change might lead to an increase in endogenous ROS levels, which results in the prevalence of the antioxidant defense system [47]. In this study, our physiological work showed that exogenous ABA resulted in an oxidative stress in alfalfa, meaning that ROS (mainly H_2_O_2_) levels accumulated rapidly. To alleviate oxidative burst, alfalfa significantly activated the antioxidant defense system using ROS detoxification of antioxidants (POD, CAT and GSH) and osmotic-adjusting substances (PRO) to maintain cellular ROS at relatively low levels. Ultimately, the ROS-activated antioxidant defense system resulted in a decrease of ion leakage and MDA levels and conferred improved abiotic stress tolerance to alfalfa (Figure 1 and Figure 2). Similar results have appeared in previous reports on the physiological effect of ABA on antioxidative defense system in maize (*Zea mays*) seedlings [48]. It is noteworthy that their study also showed that the content of MDA and ion leakage increased slightly with treatment with 100 µM or 1000 µM ABA for 24 h. Based on previous reports in *Vitis vinifera* [49] and *Arabidopsis* [50], we hypothesized that a dose dependency may exist in ABA-driven abiotic stress tolerance in alfalfa. Treatment with a low concentration of ABA (10 µM) induced an antioxidation defense response against oxidative damage; nevertheless, treatment with a high concentration of ABA (100 or 1000 µM) induced an excessive generation of ROS and led to oxidative damage in plant cells [48]. Furthermore, the chlorophyll content significantly reduced after 12 h ABA treatment in our study (Figure 1A), indicating that the photosynthesis of alfalfa is inhibited by exogenous ABA [51,52]. Photoinhibition might be a key adaptive mechanism of alfalfa in response to ABA, and enhancing the transport and hydrolysis of photosynthetic products could be the potential target for improving the abiotic stress tolerance of alfalfa [53]. Interestingly, the significant shifts of both the ion leakage and chlorophyll content occurred in leaves, indicating that ABA induces many long-distance stress signals, which move from the roots to leaves, where it regulates photosynthesis and the stability of cell membrane [54,55,56,57,58].

**Figure 10:**
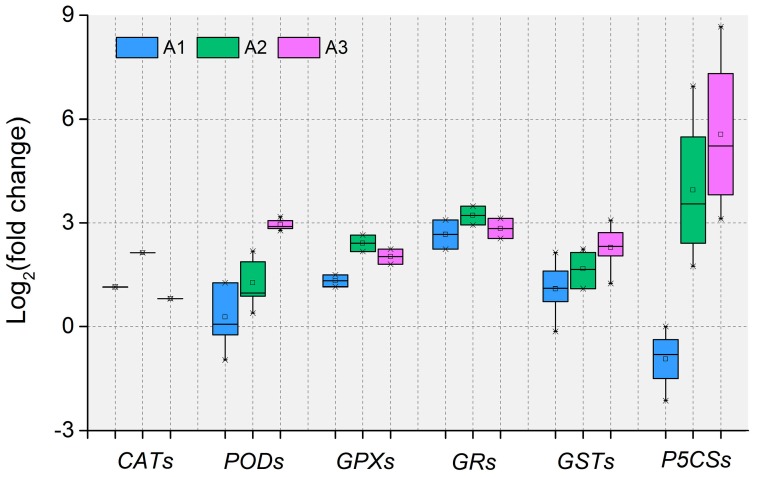
Box plot indicating the expression changes of antioxidant defense system-related DEIs. A1, A2 and A3 represent ABA treatment for 1, 3 and 12 h, respectively. The boxes show the interquartile range (IQR) between 25% (Q1) and 75% (Q3) of the values; the thick horizontal black bars are the median values, the open quadrates are average values, the whiskers define the “fence” = [Q1, Q3] +1.57×IQR, and the crosses are outliers beyond the fence.

Our transcriptome analysis also consistently revealed that the antioxidant defense system was activated at the molecular level by exogenous ABA. As is shown in Figure 10, the majority of antioxidative enzymes-related DEIs such as *CATs*, *PODs*, *glutathione peroxidases* (*GPXs*) and *glutathione reductases* (*GRs*), non-enzymatic antioxidants-related DEIs such as *glutathione S-transferases* (*GSTs*) and PRO synthetases-related DEIs such as *Δ1-pyrroline-5-carboxylate synthetases* (*P5CSs*), were significantly modulated by exogenous ABA. This is consistent with previous reports on tomato leaves under ABA treatment [18], indicating ABA induces similar gene expression of ROS scavenging-related genes both in the roots and leaves of plants. We also performed a comparative analysis with our previous study on alfalfa under cold stress [59]. Interestingly, among these antioxidant defense-related DEIs, five DEIs were co-regulated by both ABA and cold stresses, while 16 and 35 DEIs were specifically regulated under ABA and cold stress, respectively (Table S4), suggesting that both crosstalk and diversity between the ABA-induced and abiotic stress-induced responses exist in the antioxidant defense system. Overall, these results strongly suggest an important role for antioxidant defense-related DEIs in response to ABA and thus an enhancement of the capacity of protective system in the roots of alfalfa seedlings.

### 3.4. Pathogen Resistance-Related DEIs

Plants live in complex environments in which they are threatened by a vast array of pathogenic microorganisms such as fungi, oomycetes, bacteria, viruses and nematodes [60]. Unlike animals that can move and adapt to survive suboptimal conditions, plants are sessile; thus, they defeat biotic stress mainly through a combination of constitutive and inducible defense responses in the innate immune system [61]. Many of these responses are regulated by cross-communicating signal transduction pathways, within which plant hormones including SA, JA and ET fulfill central roles. SA is predominantly established local and systemic resistance to biotrophic pathogens, whereas necrotrophic pathogens are usually deterred by JA- and ET-mediated defenses [62]. In this transcriptome, a total of 8, 22 and 19 SA-, JA- and ET-related DEIs, respectively, were detected after ABA treatment, indicating that interactions occur within and between ABA and SA-JA-ET signaling networks.

**Figure 11:**
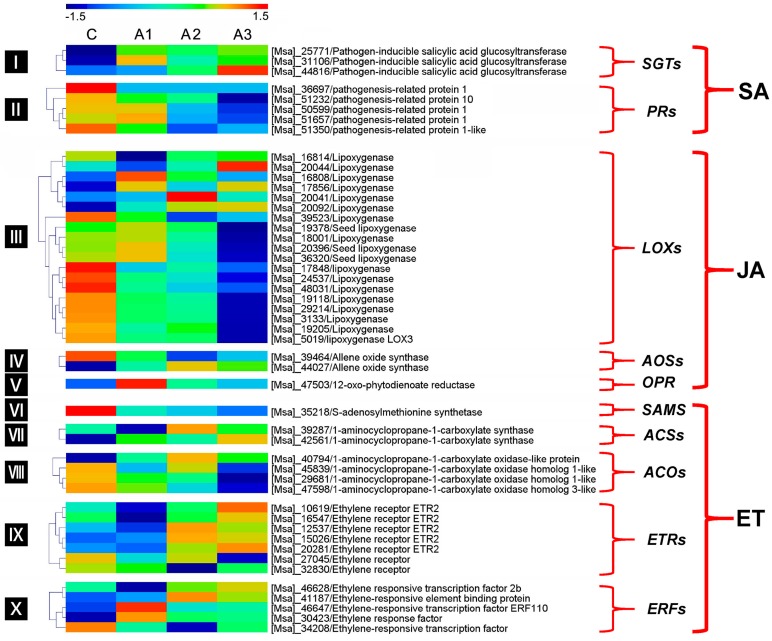
Heatmap plot of the expression levels of the key DEIs involved in SA, JA and ET regulatory pathway. C, A1, A2 and A3 represent ABA treatment for 0, 1, 3 and 12 h, respectively. The gene expression is based on the z-scores of log_2_ (FPKM) value. The red and blue colors indicate high and low expression levels, respectively.

SA glucosyltransferase (SGT) is an early disease-responsive enzyme that catalyzes the conversion of free SA into SA *O*-β-glucoside. A recent report suggested that the overexpression of *AtSGT1* in *Arabidopsis* leads to increased susceptibility to *Pseudomonas syringae* [63]. Pathogenesis-related (PR) protein, which is a critical component in the SA signaling pathway, has been reported to be strongly induced after SA treatment and enhanced resistance to pathogens in many plant species [64]. In this study, we found that all three *SGT* genes were upregulated after treatment with ABA for 12 h, whereas all five of the identified *PR* genes were greatly downregulated in group A3 after ABA deposition (Figure 11). Consequently, these results suggest that ABA deposition affects the metabolism of SA and renders alfalfa more susceptible to pathogen infection. The biosynthetic pathway of JA is regulated by several key enzymes, including the lipoxygenase (LOX), allene oxide synthase (AOS) and 12-oxo-phytodienoate reductase (OPR) [65]. A previous study found that *CaLOX1*-silenced pepper (*Capsicum annuum*) plants were more susceptible to pathogen infection [66]. In this study, 14 of the 19 *LOXs* and one of the two *AOSs* were downregulated after treatment with ABA for 12 h. The expression level of one *OPR3* was initially upregulated at A1 but later decreased to control levels at A3 (Figure 11). These results suggest that ABA has a greater negative impact than positive impact on JA-mediated resistance to pathogens in alfalfa. The major enzymes involved in ET biosynthesis include S-adenosylmethionine (SAM) synthetase (SAMS), 1-aminocyclopropane-1-carboxylic acidsynthetase (ACS) and 1-aminocyclopropane-1-carboxylic acid oxidase (ACO) [67]. SAMS catalyzes the synthesis of SAM from ATP and L-methionine. The biosynthesis of ET from SAM is catalyzed sequentially by ACS and ACO [68]. Additionally, ET receptors (ETRs) and ERF TFs are vital to the ET signaling pathway [18]. In this study, all the genes involved in ET biosynthesis and signaling mentioned above were identified; 9 of these genes were upregulated, whereas 10 were downregulated during the ABA treatment (Figure 11). These data thus favor a scenario in which ET acts as a two-faced defense regulator in alfalfa [62].

Furthermore, there is much evidence concerning the implications of a large number of genes encoding phenylalanine ammonia-lyase (PAL), β-1,3-glucanase (GLU) and heat shock protein 90 (HSP90) are believed to be involved in biotic stress responses and resistances in many plant species [69,70]. Nearly all of these DEIs were stably downregulated after treatment with ABA for 12 h (Figure S4), further suggesting that ABA negatively affects alfalfa in response to pathogens and these pathogen-related enzymes and proteins may act as the downstream regulators of hormone-signaling pathways in alfalfa root tips. However, a transcriptome analysis of tomato leaves treated with ABA (7.58 μM) solutions for 24 h largely up-regulated the genes related to biotic stresses (such as *PALs* and *GLUs*), indicating that ABA has the potential to promote pathogen resistance in tomatoes [18]. This may be due to the different plant material, ABA concentration and ABA treatment time used in our study. Taken together, ABA interacts antagonistically or synergistically with SA, JA and ET in various ways, and ABA may primarily act as a negative regulator in alfalfa biotic stress resistance to both biotrophic and necrotrophic pathogens.

## 4. Materials and Methods

### 4.1. Plant Materials and Growth Conditions

Alfalfa seeds of Zhongmu No. 1 were kindly provided by Prof. Qingchuan Yang from the Institute of Animal Sciences, Chinese Academy of Agricultural Sciences. The seeds were surface sterilized and then placed on sterilized filters that were moistened with distilled water in inverted square Petri dishes at 22 °C. After 5 days of germination, 48 seedlings with uniform taproot lengths were alternately sown in 96-well plates supported by a plastic container and hydroponically grown in half-strength Murashige and Skoog (1/2 MS) nutrient solution (pH = 5.8). The seedlings were then grown in a controlled-environment chamber for 1 week under the following conditions: A temperature of 22 °C, a daily photoperiod of 16 h light/8 h dark, a flux density of 180 µmol m^−2^s^−1^ and a relative humidity of 80%. Solutions were changed once every 2 days to maintain constant nutrient concentrations.

### 4.2. ABA Treatment

Following the growth in the controlled-environment chamber, the 12-day-old seedlings were separated into four groups, including three ABA-treatment time point groups (A1, A2 and A3) in a 1/2 MS nutrient solution containing 10 µM ABA (pH = 5.8) and one control (C) group. Three biological replicates were included for each treatment time point, including the C group. To harvest the treated seedlings across all treatments at the same time, the ABA treatment for the different treatment groups was staggered. The ABA treatment was started at 9 am on the 12-h treatment sample, at 6 pm on the 3-h treatment sample and then 8 pm on the 1-h treatment sample, and these ABA-treated samples were all harvested at 9 pm together with a non-treated control sample. The trifoliate leaves (a pool of 10 different leaves) and root tips (approximately 1.5 cm in length; a pool of 20 different root tips) were harvested for physiological analysis. Other root tips were harvested for sequencing analysis; those root tips were flash-frozen in liquid nitrogen and then stored at −80°C.

### 4.3. Determination of Physiological Characteristics

All 12 samples (three biological replicates for each of the four treatment groups) were immediately assessed using eight physiological indices: for the leaves, the chlorophyll content and electrolyte leakage were determined, respectively, as described previously [71,72]. For the roots tips, lipid peroxidation via MDA; the production of ROS, such as H_2_O_2_; the activity of antioxidative enzymes, such as POD and CAT; the content of non-enzymatic antioxidants, such as GSH; and osmotic-adjusting substances, such as PRO, were determined using Comin Biochemical Test Kits (MDA-2-Y, H_2_O_2_-2-Y, POD-2-Y, CAT-2-Y, GSH-2-W and PRO-2-Y, respectively; Cominbio, Suzhou, China) in accordance with the manufacturer’s instructions. In brief, the MDA content was assayed based on the thiobarbituric acid-reactive substance assay according to the method of Castrejón and Yatsimirsky (1997) [73]; the H_2_O_2_ content was determined by monitoring the absorbance of titanium-peroxide complex at 415 nm according to the method described by Men et al. (2018) [74]; the POD activity was assayed based on the detection of the absorbance of the product at 470 nm in the reaction system according to the method of Toivonen and Sweeney (1998) [75]; the CAT activity was determined by measuring the rate of decomposition of H_2_O_2_ (ε = 39.4 mM^−1^cm^−1^) at 240 nm as described by Men et al. (2018) [74]; the GSH content was measured by detecting the absorbance of 5,5′-Dithiobis-(2-nitrobenzoic acid)-GSH complex at 412 nm according to the method of Men et al. (2018) [74] ; and the PRO content was measured using the acid ninhydrin method described by Vieira et al. (2010) [76].

### 4.4. cDNA Library Preparation, Sequencing, Assembly and Annotation

The RNA extractions, as well as the quality and quantity measurements of all 12 alfalfa samples, were performed as previously described [77]. For RNA-seq, the total RNA from all 12 alfalfa samples was separately used to prepare cDNA libraries and sequenced on a BGISEQ-500 RS platform at BGI Shenzhen [59]. After quality control checks, the high-quality clean reads were separated from the raw data and then mapped to the “MSA” reference full-length transcriptome database using Bowtie2 software, which were obtained from our previous study of transcriptome sequencing performed by PacBio Iso-Seq (SRR7091350–53).

All full-length transcripts were subsequently annotated into three public databases, including the NCBI Nr, GO and KEGG databases. Additionally, the gene expression level was quantified by the RSEM software package [78] and was normalized by the FPKM method [79].

### 4.5. qRT-PCR Analysis

The total RNA of all 12 alfalfa samples used for the RNA-Seq analysis was also used to make cDNAs for qRT-PCR validation. In brief, the single-strand cDNAs used for qRT-PCR were synthesized from one µg of the total RNA using FastQuant RT Kit (with gDNase) (Tiangen Biotech, Beijing, China) in accordance with the manufacturer’s instructions. The qRT-PCR analysis was performed using 2xSG Fast qPCR Master Mix (Sangon Biotech, Shanghai, China) on a 7500 Fast Real-time PCR system (Applied Biosystems, Foster City, CA, USA) under the following parameters: 95 °C for 30 s, 40 cycles of 95 °C for 5 s and 60 °C for 30 s. Gene-specific primers for qRT-PCR were designed via DNAMAN software (Lynnon BioSoft, Vandreuil, Quebec, Canada) and are shown in Table S5. Three technical replicates were performed for each sample. The Ubiquitin gene (MSAD_296100.t1) of alfalfa was used as the housekeeping gene and the relative gene expression levels were calculated according to the 2^−∆∆*C*t^ method.

### 4.6. DEIs Analysis

Based on the average FPKM values in each treatment, differential expression between the treatment group and control group was assessed using the NOISeq package [80]. Both the absolute values of the fold change ≥4 and the divergence probability ≥0.8 were used as thresholds to identify significant DEIs. The cluster analysis and expression pattern assessment were performed by MEV 4.9 software via the hierarchical clustering and the K-means clustering methods [81], respectively. The GO and KEGG pathway enrichment analyses for DEIs were performed using agriGO 2.0 [82] and KOBAS 3.0 [83], respectively. TFs were predicted into different families using the PlantTFDB [84], and the cluster analysis for TFs was conducted by the SOTA using the MEV4.9 software [81].

## 5. Conclusions

In this study, we presented a comprehensive transcriptome analysis of alfalfa roots under a prolonged time-course for ABA treatment. These sequences were assembled into 50,742 isoforms, with an average length of 2541 bp. Next, a total of 4944 ABA-regulated DEIs were identified and analyzed for their potential role in the response to abiotic stress and biotic stress using clustering, GO and KEGG enrichment analysis. These DEIs were mainly involved in plant hormone signal transduction, transcriptional regulation, antioxidative defense and pathogen immunity. Furthermore, by analyzing the expression pattern of the related genes during a 12-h ABA treatment, our study provides support for the idea that in alfalfa differential molecular mechanisms exist between abiotic and biotic stress responses. Overall, a detailed investigation of the core pathways and candidate genes was provided in this study, which deepens the understanding of the molecular mechanisms underlying alfalfa responses to ABA and offers potential targets for the improvement of forage crops via breeding.

## Figures and Tables

**Table 1:** Distribution of differentially expressed TFs.

TF Family	A1	A2	A3	Total
ABI3VP1	0	1	0	1
AP2-EREBP	3	6	3	9
ARF	2	4	3	6
bHLH	1	1	1	3
bZIP	11	9	7	12
C2C2-Dof	1	1	0	1
C2H2	1	1	0	1
C3H	2	1	1	4
E2F-DP	0	0	1	1
EIL	2	1	1	2
FAR1	0	1	0	1
G2-like	0	0	1	1
GRAS	1	3	1	3
HB	0	1	0	1
HSF	0	1	1	2
LOB	1	0	0	1
MADS	0	0	1	1
mTERF	0	0	1	1
MYB	4	6	6	9
NAC	3	3	2	3
TAZ	0	1	0	1
TIG	3	3	1	5
Trihelix	7	1	0	8
TUB	2	1	1	3
WRKY	0	0	2	2
Total	44	46	34	82

Note: A1, A2 and A3 represent ABA treatment for 1, 3 and 12 h, respectively.

## Supplementary Materials

Supplementary materials can be found at http://www.mdpi.com/1422-0067/20/1/47/s1.

## Abbreviations

ABA	Abscisic acid
ABF	ABA-responsive elements binding factor
CAT	Catalase
DEI	Differentially expressed isoform
ET	Ethylene
FPKM	Fragments per kilobase per million fragments mapped
GO	Gene Ontology
GSH	Glutathione
H_2_O_2_	Hydrogen peroxide
JA	Jasmonate
KEGG	Kyoto Encyclopedia of Genes and Genomes
MDA	Malonaldehyde
MEV 4.9	MultiExperiment Viewer 4.9
NCBI	National Center for Biotechnology Information
NGS	Next-generation sequencing
Nr	Non-redundant protein sequences
POD	Peroxidase
PP2C	Protein phosphatase 2C
PRO	Proline
PYL	Pyrabactin resistance 1-like
qRT-PCR	Quantitative real-time polymerase chain reaction
RNA-Seq	RNA sequencing
ROS	Reactive oxygen species
SA	Salicylic acid
SnRK2	Sucrose nonfermenting1-related protein kinase 2
SOTA	Self-Organizing Tree Algorithm
TF	Transcription factor
