# Supplementary material for: Comparative Transcriptomic and Physiological Analyses of Medicago sativa L. Indicates that Multiple Regulatory Networks Are Activated during Continuous ABA Treatment
Source: Int J Mol Sci. 2018 Dec 22;20(1):47. doi: 10.3390/ijms20010047 (PMC6337461; doi:10.3390/ijms20010047)
Supplement: Supplementary file 1 [file ijms-20-00047-s001.zip › Supplementary materials/Supplementary Figures S1-S4.pdf]

Comparative transcriptomic and physiological analyses of *Medicago sativa* L. indicates that multiple regulatory networks are activated during continuous ABA treatment

Dong Luo, Yuguo Wu, Jie Liu, Qiang Zhou, Wenxian Liu, Yanrong Wang, Qingchuan Yang, Zengyu Wang and Zhipeng Liu

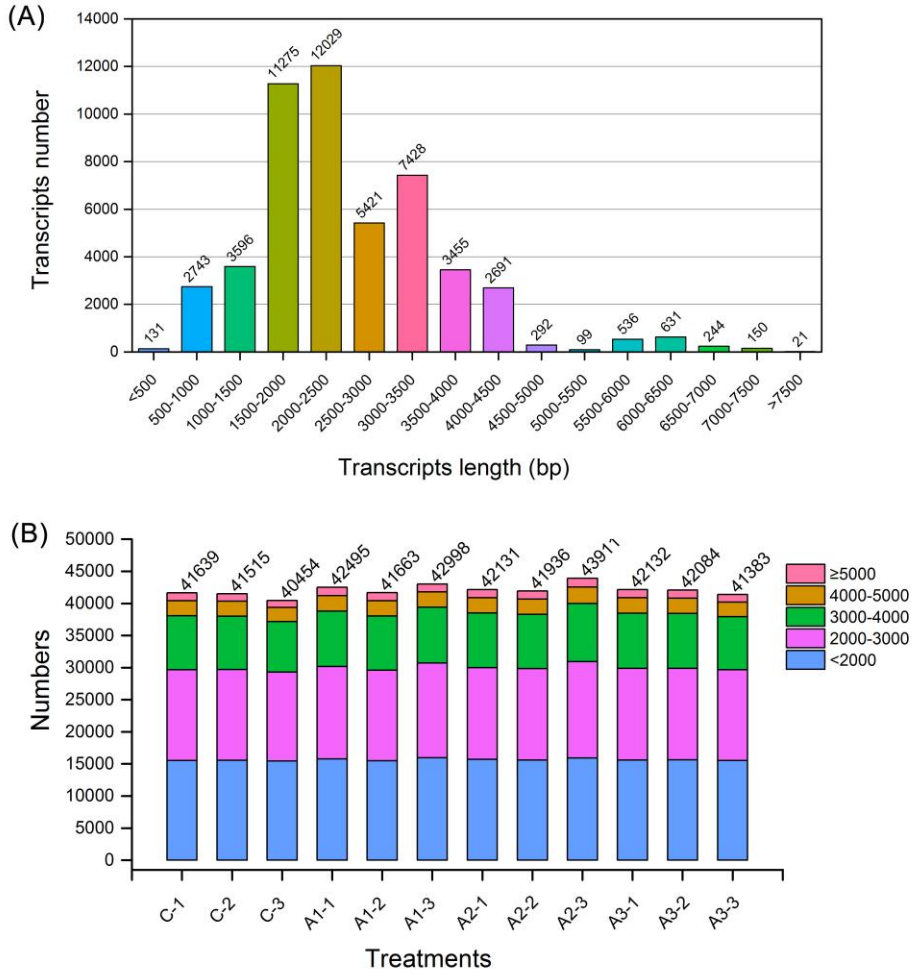

Figure S1. The quality of the assembled isoforms. C, A1, A2 and A3 represent ABA treatment for 0, 1, 3 and 12 h, respectively.

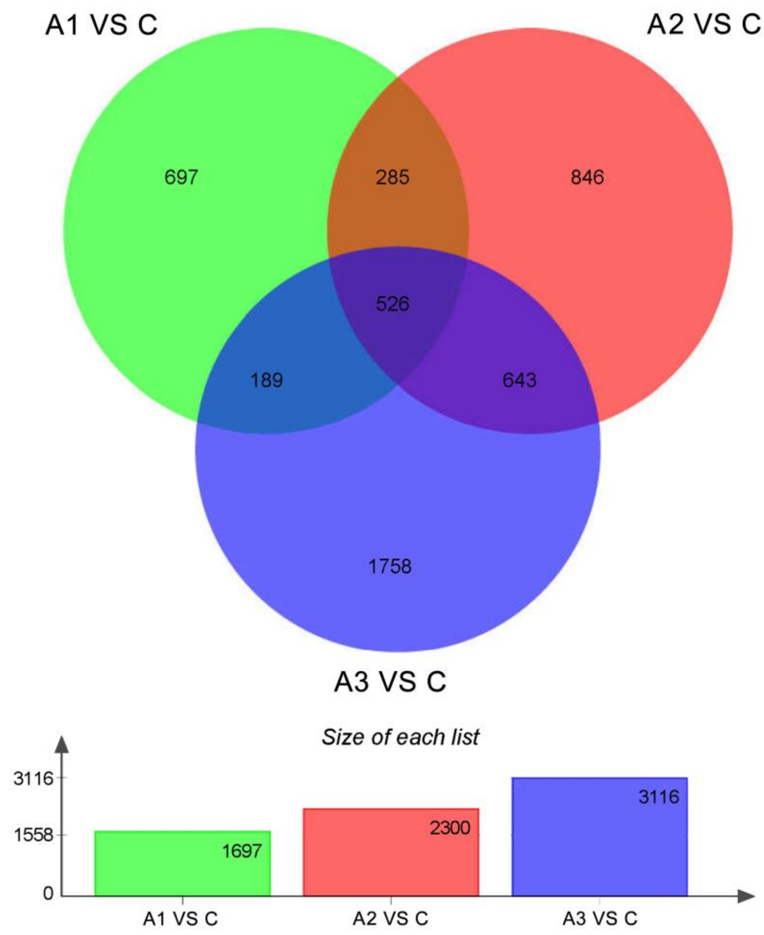

Figure S2. The number of DEIs expressed at one ABA-treated time point and at overlapping time points compared with the control. C, A1, A2 and A3 represent ABA treatment for 0, 1, 3 and 12 h, respectively.

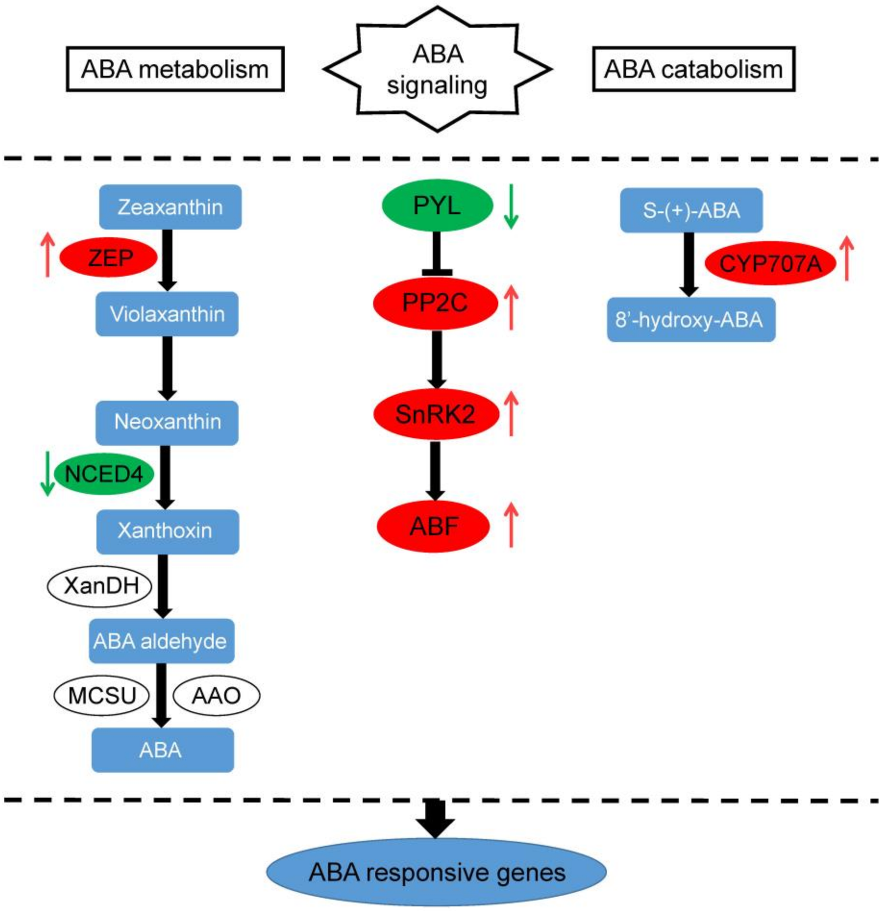

Figure S3. Models describing the ABA regulatory pathways regulated by exogenous ABA in alfalfa. Exogenous ABA that affect endogenous ABA regulates ABA signal pathway core components. Under ABA treatment, ABA key biosynthesis and catabolism transcripts were dynamically regulated. Meanwhile, expression levels of ABA-bound receptor PYLs were conformably downregulated, while the protein kinases of PP2Cs and SnRK2s were uniformly upregulated. The downstream ABA-dependent TFs, such as ABFs, were then activated, which promote most downstream ABA-responsive gene transcription in most cases.

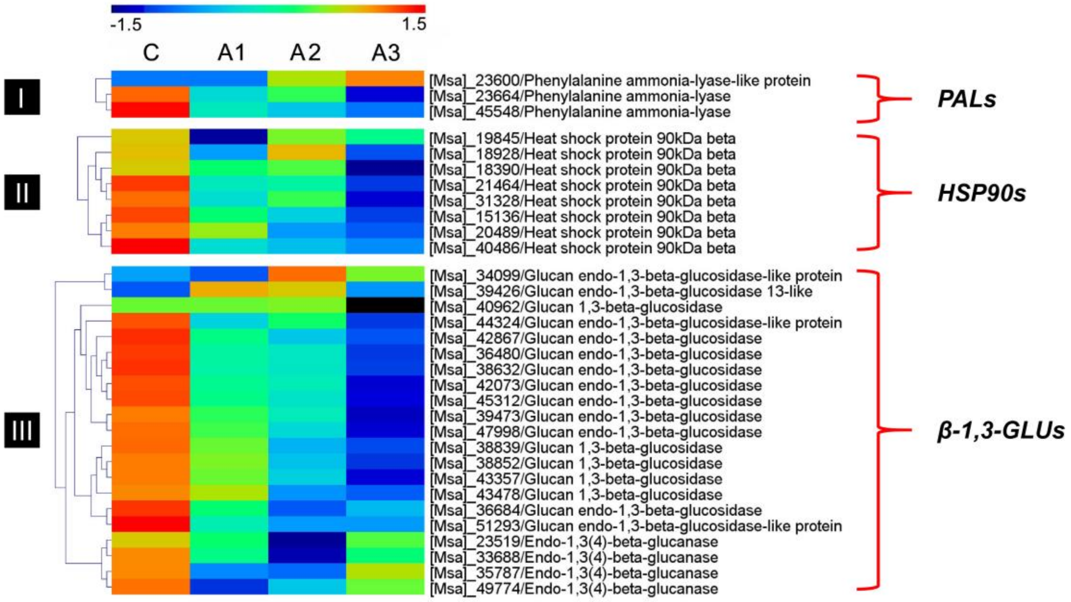

Figure S4. Heatmap plot of the expression levels of the key DEIs encoding PAL, β-1,3-GLU and HSP90. C, A1, A2 and A3 represent ABA treatment for 0, 1, 3 and 12 h, respectively. The gene expression is based on the z-scores of log_2 (FPKM) value. The red and blue colors indicate high and low expression levels, respectively.
